# Coexistence of a novel STRN-ALK, NBEA-ALK double-fusion in an ovarian malignant mesothelioma patient: a case report and review

**DOI:** 10.3389/fonc.2023.1156329

**Published:** 2023-04-21

**Authors:** Xiao Wu, Qi Wang, Xiaohu Xu

**Affiliations:** Department of Integrated Traditional Chinese and Western Medicine, Tongji Hospital, Tongji Medical College, Huazhong University of Science and Technology, Wuhan, China

**Keywords:** primary ovarian mesothelioma, anaplastic lymphoma kinase gene (ALK), STRN, neurobeachin (NBEA), case report

## Abstract

Primary ovarian mesothelioma (POM) is a rare malignant tumor with poor prognosis. Although anaplastic lymphoma kinase gene (ALK) double-fusion partners have been found in various tumors, it is rarely reported in mesothelioma. In this article, we describe the coexistence of a novel STRN-ALK, neurobeachin (NBEA)-ALK double-fusion in a patient with primary ovarian mesothelioma. A 30-year-old woman was found to have pelvic masses for more than a year. Color Doppler ultrasound showed mixed mass in the left ovary and multiple solid masses in the right ovary. the patient underwent laparoscopic surgery, including total hysterectomy, bilateral salpingo-oopherectomy, pelvic lymph node and abdominal aortic lymph node resection, omentum resection and abdominal focus resection. Pathologic examination revealed bilateral ovarian malignant mesothelioma and no evidence of malignancy in the resected bilateral round/broad ligaments, bilateral parametrial tissues, vaginal stump, bilateral fallopian tubes, pelvic and paraaortic lymph nodes. Immunohistochemistry showed that it was positive for Calretinin, VIM, WT1, PAX8, mesothelin, CK5/6, PCK, CK7, MLH1, PMS2, MSH2, MSH6, weakly positive for BAP1, while being negative for Napsin A, P504S, CEA, D2-40, GATA3. The sequencing analysis identified STRN-ALK (intron3:intron19) and NBEA-ALK (intron1:intron16) double-ALK fusion. To the best of our knowledge, this is the first report that a novel NBEA-ALK and EML4-ALK coexist in one patient with POM. The patient has completed 6 cycles of continuous chemotherapy and is in stable condition. Whether ALK inhibitors can bring promising benefits to POM patients in the future deserves further study.

## Introduction

Mesothelioma is a rare clinical mesothelial cell-derived tumor with a high degree of malignancy, which can involve the pleura, peritoneum, pericardium, ovary and testis. It is more common to originate from pleura and peritoneum. However, mesothelioma originating from ovary is extremely rare (1). According to a survey of over a 24-year period in UK, primary ovarian mesothelioma only accounts for 0.03% of mesothelioma-related deaths (2). In 1983, Addis et al. reported a 67-year-old white woman with a large tumour of the left ovary, complaining of nausea, upper abdominal pain and biliary regurgitation, and was diagnosed as papillary mesothelioma of ovary (3). Sun et al. reported that a 50-year-old woman found a hypermetabolic mass in the left ovary by ^18^F-FDG PET/CT, and was diagnosed as ovarian mesothelioma pathologically after biopsy in 2019 (4).Another study reported a female patient with ovarian mesothelioma who underwent surgery complemented by adjuvant chemotherapy, with complete radiation response and a disease-free survival time of 1 year (5). In Clement et al’s study, 9 cases of mesothelioma presenting as ovarian masses were described, of which at least 2 cases were to be primary in the ovary (6). According to the latest review, only 19 cases of ovarian mesothelioma were reported (7).

Primary ovarian mesothelioma has the same cell origin as other epithelial ovarian tumors, so it is difficult to distinguish malignant mesothelioma from malignant ovarian tumor and metastatic tumor by imaging examination. Immunohistochemistry plays an important role in identifying neoplastic phenotype by introducing sensitive and specific antibodies. Next-generation sequencing (NGS) provides more comprehensive details for genome changes of cancer patients, and has advantages in identifying therapeutic targets and rare gene mutations. Anaplastic lymphoma kinase (ALK) is a transmembrane receptor tyrosine kinase, which is considered to be the proto-oncogene responsible for encoding receptor tyrosine kinase (8). The ALK gene mutation was originally discovered by NPM1-ALK fusion in anaplastic large cell lymphoma, which is the first gene mutation found in human cancer (9). Since then, different ALK fusion partners have been found in various tumors, including lymphoma, neuroblastoma and non-small cell lung cancer (NSCLC) (10). As an ALK fusion partner, STRN has been reported in some published articles, such as NSCLC, renal cancer, leukemia, breast cancer and thyroid cancer (11). Neuroglobulin (NBEA), as a key regulator of postsynaptic neurons, was initially found to be a possible novel tumor suppressor gene in multiple myeloma, but recently it was found that it showed a high mutation rate in gastric cancer, while its expression decreased in oropharyngeal squamous cell carcinoma (12). Up to now, only one paper reported that a new dual fusion protein NBEA-ALK and EML4-ALK existed in a lung adenocarcinoma patient (13). However, double ALK fusion mutation in ovarian mesothelioma has not been reported. In this article, we introduce a case of ovarian mesothelioma, which has a new neurotoxin (NBEA)-ALK and STRN-ALK double fusion.

## Case presentation

A 30-year-old female was admitted to our hospital with a pelvic mass existing about one year in June 2022. She had a history of cesarean section in a local hospital and no pelvic masses were found during the operation in April 2018 and August 2020, respectively. She had no history of asbestos or radiation exposure. The last menstrual period was approximately the end of May 2022. On examination, her abdomen was soft without tenderness, a hard mass (3×3cm) was palpable in her left lower abdomen and a cystic mass (6×4cm) was palpable in her right lower abdomen. Color Doppler ultrasound showed mixed mass in the left ovary (1.9x1.6cm anechoic area contains a 1.3x0.7cm hyperechoic area and a 1.8x1.0cm hypoechoic area), and multiple solid masses in the right ovary (5.9x3.6cm anechoic area contains multiple hypoechoic areas, one of which is 3.3x2.6cm in size) on June 27, 2022.

After admission, laboratory tests were performed, including blood routine, urine routine, liver function, kidney function, electrolytes, blood lipids, and coagulation function. No obvious abnormalities were found. The levels of tumor markers were shown as follows: cancer antigen-125 (CA-125): 30.5 U/mL; human epididymis protein 4 (HE4): 78.7 pmol/L; carcinoembryonic antigen (CEA): 1.05 ng/mL; squamous cell carcinoma antigen (SCC): 1.1 ng/mL; cancer antigen 72-4(CA 72-4): 3.85 U/mL; β-hCG<0.10 mIU/mL; neuron-specific enolase (NSE): 10.59 ug/L; alpha-fetoprotein(AFP): 1.55 ng/ml; carbohydrate antigen-153 (CA-153): 10.9 U/mL; carbohydrate antigen -199 (CA-199): 8.27 U/mL.

**Figure 1:**
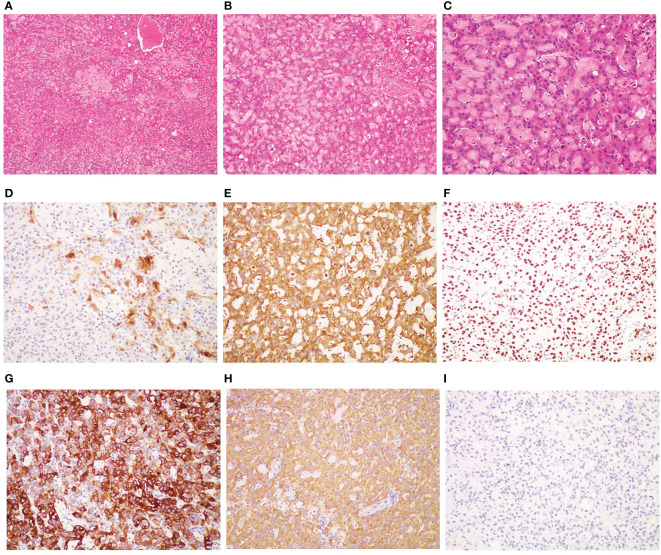
Pathological and immunohistochemical examination results. **(A)** hematoxylin-eosin staining(HE), magnification ×40; **(B)** HE, mangnification×100; **(C)** HE; magnification×200; **(D)** Calretinin positivity, magnification×200; **(E)** VIM positivity, magnification×200;**(F)** WTI positivity, magnification×200; **(G)** CK5/6 positivity, magnification×200; **(H)** PCK positivity, magnification×200; **(I)** CEA negativity, magnification×200.

After obtaining written informed consent, the patient underwent laparoscopic surgery, including total hysterectomy, bilateral salpingo-oopherectomy, pelvic lymph node and abdominal aortic lymph node resection, omentum resection and abdominal focus resection on July 4, 2022. Pathologic examination revealed bilateral ovarian malignant mesothelioma and no evidence of malignancy in the resected bilateral round/broad ligaments, bilateral parametrial tissues, vaginal stump, bilateral fallopian tubes, pelvic and paraaortic lymph nodes. Immunohistochemistry showed that it was positive for Calretinin, VIM, WT1, PAX8, mesothelin, CK5/6, PCK, CK7, MLH1, PMS2, MSH2, MSH6, weakly positive for BAP1, while being negative for Napsin A, P504S, CEA, D2-40, GATA3 (**Figure 1**). The Ki67 index was 10%. Therefore, it was diagnosed as primary ovarian mesothelioma.

**Figure 2:**
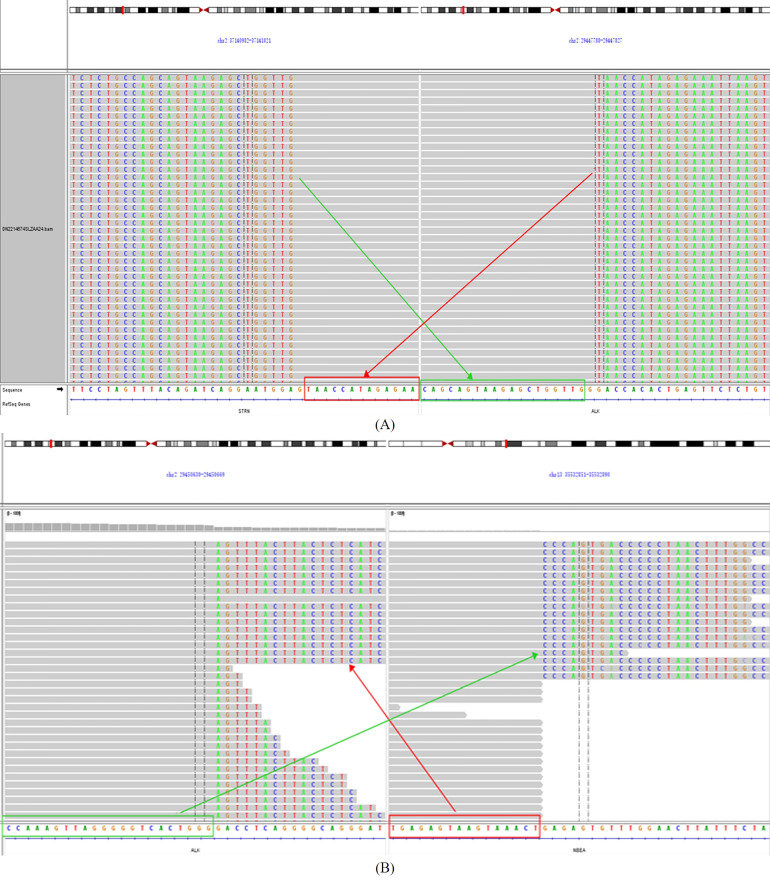
**(A)** identification of the STRN-ALK fusion; **(B)** identification of the NBEA-ALK fusion.

In the meantime, in the absence of standard posterior-line treatment, after obtaining the consent of the patient, we submitted the neoplasm samples of the patient for NGS analysis in order to find potential therapeutic targets. Gene analysis of the neoplasm samples was undertaken by YuceBio Technology Co., Ltd (Shengzhen, China) using Illumina NovaSeq 6000 platform (Illumina, San Diego, CA). The sequencing analysis identified STRN-ALK (intron3:intron19) and NBEA-ALK (intron1:intron16) double-ALK fusion (**Figure 2**). In STRN-ALK fusion, intron 3 of the STRN gene and intron 19 of the ALK gene were broken and rearranged, and the mutation abundance is 27.74% (**Table 1**). In NBEA-ALK fusion, intron 1 of the NBEA gene and intron 16 of the ALK gene were broken and rearranged, and the mutation abundance is 49.26% (**Table 1**). This double fusion mutation has never been reported in primary ovarian mesothelioma.

**Table 1:** Gene fusion mutation result.

5'-terminal gene	5'-terminal chromosome	5'-terminal breakpoint	3'-terminal gene	3'-terminal chromosome	3'-terminal breakpoint	Mutation abundance
STRN NM_003162	chr2	intron3	ALK NM_004304	chr2	intron19	27.74%
NBEA NM_015678	chr13	intron1	ALK NM_004304	chr2	intron16	49.26%

**Figure 3:**
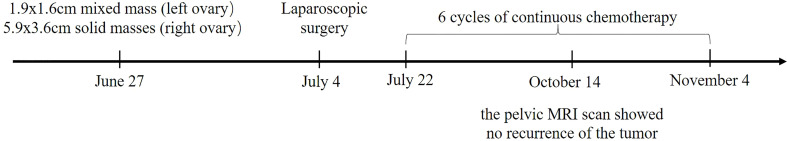
Timeline of clinical events.

On July 22, she started the first cycle of chemotherapy, with injection pemetrexed (500mg/m^2^) and injection nedaplatin (80mg/m^2^), which was repeated every 3 weeks. On August 31, 2022, the disease was assessed as stable by pelvic MRI scan, and chemotherapy continued. As recently as October 14, 2022, the pelvic MRI scan showed no recurrence of the tumor, indicating that it was still stable. To date, the patient has completed 6 cycles of continuous chemotherapy, and no obvious adverse symptoms have been observed. The patient’s condition is stable, and the follow-up work is still in progress. The treatment timeline of this patient is illustrated in **Figure 3**.

## Discussion

The diagnosis of primary ovarian mesothelioma often requires intraoperative tumor morphology and ovarian involvement, combined with pathological and immunohistochemical examination. Firstly, it needs to be distinguished from primary peritoneal malignant mesothelioma. Malignant peritoneal mesothelioma generally does not involve bilateral ovaries, and the morphology and size of ovaries are normal. During the operation, it was found that the present patient’s tumor involved both ovaries, but there was no tumor on the surface of abdominal wall and peritoneum, which ruled out the possibility of peritoneal malignant mesothelioma and considered primary ovarian tumor. Secondly, there is no obvious tumor tissue in colon, liver, spleen, diaphragm, liver and kidney recess, spleen and kidney recess during the operation, and there was no mass shadow in lung CT examination, which ruled out the possibility of metastasis. Last but not least, immunohistochemical positive markers commonly used in malignant mesothelioma include Calretinin, Cytokeratin5/6(CK5/6), Vimentin(VIM), WT-1, and Podoplanin (D2-40), among which the highest specificity is Calretinin, and the highest sensitivity is Vimentin. The Associations of Directors of Anatomic and Surgical Pathology (ADASP) does not recommend specific markers, but it is recommended that at least two positive mesothelial markers (calretinin, CK5/6, D2-40 and WT-1) be used in combination with at least two or more negative mesothelial markers (CEA, TTF-1, Ber-EP4 and MOC-31) (14, 15). At present, it is generally believed that the diagnosis of malignant mesothelioma requires the positivity of Calretinin, Vimentin and PCK, and the negative of monoclonal CEA (15). Immunohistochemical results of the present case showed that Calretinin, Vimentin, WT-1, CK5/6 and PCK were positive and CEA was negative, which met the diagnostic requirements of mesothelioma. Therefore, the patient was finally diagnosed as primary ovarian mesothelioma.

The treatment of mesothelioma includes surgery, radiotherapy, systemic chemotherapy and intraperitoneal chemotherapy. At present, radical resection and postoperative adjuvant chemotherapy are the main treatment methods for mesothelioma. The combination of platinum and antifolates(e.g.pemetrexed)is the most commonly used chemotherapy regimen (16). Addition of antiangiogenic drugs (bevacizumab) to pemetrexed plus cisplatin can significantly improve the OS of malignant pleural mesothelioma, which is expected to be the first-line standard care (17). In recent years, immunotherapy has brought new hope for the treatment of solid malignant tumors. Checkmate 743 study proved for the first time that double immunotherapy can improve the survival of patients with advanced malignant pleural mesothelioma (18). An exciting news is that FDA recently approved nivolumab plus ipilimumab for the first-line treatment of advanced mesothelioma, which is an important progress for the treatment of this disease (19). With the application of genomic analysis technology in recent years, it is helpful to provide potential targets for the treatment of mesothelioma. The reported mutation genes related to malignant mesothelioma include CDKN2A/2B, BAP1, NF2 and LAST2, and the targeted therapeutic drugs for these genes is still in the research stage (20). In addition, some studies have evaluated the efficacy of molecular targeted drugs on mesothelioma in recent years. It has been reported that the epithelial growth factor receptor (EGFR) is overexpressed between 44 and 97% of mesothelioma patients, but some studies show that gefitinib and erlotinib have no better effect than chemotherapy (21).

ALK gene recombination, as a tumor driver, promotes the proliferation of tumor cells by activating downstream signal pathways including STAT3 (signal transducer and activator of transcription-3), PI3K (phosphatidylinositol 3-kinase), mTOR (mammalian target of rapamycin) and MAPK (mitogen-activated protein kinase) (10). ALK fusion is a well-studied carcinogenic alteration in NSCLC. It is the second most common driver genomic alteration, after EGFR gene mutations (22). Chromosome rearrangement involving ALK has been identified as a drug-targeted gene change in NSCLC patients. At present, various ALK inhibitors such as crizotinib, seretinib, aletinib, and lorlatinib, are used as the first-line treatment for advanced NSCLC with positive ALK (23). Several types of ALK fusion have been reported in NSCLC patients, among which EML4-ALK fusion is the most common ALK rearrangement (24). The STRN-ALK fusion is an extremely rare ALK rearrangement that was first reported in NSCLC patients using RNA sequencing in 2017 (25). STRN is located in the very same short arm of chromosome 2 as ALK and EML4. As a fusion partner, STRN has been detected in NSCLC, renal carcinoma, leukemia, breast cancer and thyroid cancer (22). However, STRN-ALK fusion is even rarer in malignant mesothelioma. An article reported a 9-year-old child with STRN-ALK fusion malignant peritoneal mesothelioma (MPM) did not benefit from crizotinib (26). Another article reports a 13-year-old girl with MPM with STRN-ALK gene fusion, whose clinical symptoms improved significantly after treatment with seretinib (27). Another article describes a case of a 5-year-old patient with MPM driven by the STRN-ALK rearrangement who had complete remission for more than 3 years after receiving crizotinib combined with cisplatin and gemcitabine chemotherapy (28). A recent literature reported that two malignant peritoneal mesothelioma patients with STRN-ALK fusion mutation had pelvic tumor and liver metastasis after treatment with crizotinib, which was improved after the use of brigatinib (29). Up to now, there is no report of POM with STRN-ALK fusion (30). Surprisingly, the NBEA-ALK fusion mutation was also present in this case. NBEA is a family protein with BEACH domain, which is dedicated to vesicle transport (31). It has been proven to be a gene signature for predicting the prognosis of gastric cancer, and it interacts with NOTCH1 as a transcription regulator in the nucleus (12). Other studies suggested that NBEA gene might be related to the increased bladder tumour size (32), while NBEA expression decreases in oropharyngeal squamous cell carcinomas (33). There is only one case reported that coexistence of a novel NBEA-ALK, EML4-ALK double-fusion is sensitive to alectinib in a lung adenocarcinoma patient (13). NBEA-ALK fusion of malignant mesothelioma has never been reported previously.

To the best of our knowledge, this is the first report that a novel NBEA-ALK and EML4-ALK coexist in one patient with POM. In addition, different from previous reports, the present case shows STRN-ALK (intron3:intron19) and NBEA-ALK (intron1:intron16) double-ALK fusion, with mutation abundance of 27.74% and 49.26% respectively (13). The patient had received 6 cycles of first-line pemetrexed combined with nedaplatin chemotherapy, and no tumor recurrence or metastasis was found till now. Therefore, targeted drug therapy was not given. Considering the rare primary ovarian mesothelioma, whether patients with two rare ALK rearrangements can benefit from ALK inhibitors needs further attention in the future.

In conclusion,POM is a highly malignant and rare primary malignant tumor derived from ovarian mesothelial cells. The rarity of this tumor and its difficulty in differentiating from ovarian cancer or peritoneal mesothelioma easily lead to misdiagnosis or missed diagnosis. Therefore, it is very important to report such rare cases to raise the awareness of this tumor. To our knowledge, this is the first report that a novel NBEA-ALK, STRN-ALK double-ALK fusion in a POM patient. Genome analysis technology is helpful to provide potential targets for the treatment of mesothelioma. The experience of treating the same mutation target in other tumors can provide some guidance for the treatment of this rare disease. Of course, whether ALK inhibitors can bring promising benefits to POM patients in the future deserves further study.

## Data availability statement

The original contributions presented in the study are included in the article/supplementary material. Further inquiries can be directed to the corresponding author.

## Ethics statement

The studies involving human participants were reviewed and approved by The Clinical Trial Ethics Committee of Tongji Hospital, Tongji Medical College, Huazhong University of Science and Technology. The patients/participants provided their written informed consent to participate in this study. Written informed consent was obtained from the individual(s) for the publication of any potentially identifiable images or data included in this article.

## Author contributions

QW designed the idea. XW collected the data. XHX wrote the manuscript. All authors read and approved to submit the report for publication. All authors contributed to the article and approved the submitted version.
